# Varying Analgesic Effectiveness of Systemic and Central Intrathecal Administration of Cyclooxygenase‐2 Inhibitors in Different Phases of Osteoarthritic Pain in Rats

**DOI:** 10.1002/ejp.70068

**Published:** 2025-07-04

**Authors:** Chun‐Sung Sung, Shi‐Ying Huang, Hao‐Jung Cheng, Sung‐Chun Lin, Zong‐Sheng Wu, Zhi‐Kang Yao, Nan‐Fu Chen, Yen‐Hsuan Jean, Zhi‐Hong Wen

**Affiliations:** ^1^ Division of Pain Management, Department of Anesthesiology Taipei Veterans General Hospital Taipei Taiwan; ^2^ School of Medicine National Yang‐Ming Chiao Tung University Taipei Taiwan; ^3^ College of Ocean Food and Biological Engineering Jimei University Xiamen China; ^4^ Department of Orthopedic Surgery Pingtung Christian Hospital Pingtung Taiwan; ^5^ Department of Marine Biotechnology and Resources National Sun Yat‐Sen University Kaohsiung Taiwan; ^6^ Department of Orthopedic Surgery Kaohsiung Veterans General Hospital Kaohsiung Taiwan; ^7^ Division of Neurosurgery, Department of Surgery Kaohsiung Armed Forces General Hospital Kaohsiung Taiwan; ^8^ National Museum of Marine Biology and Aquarium Pingtung Taiwan; ^9^ Institute of Biopharmaceutical Sciences National Sun Yat‐Sen University Kaohsiung Taiwan

**Keywords:** anterior cruciate ligament transection, celecoxib, cyclooxygenase‐2, etoricoxib, mechanical allodynia, osteoarthritis

## Abstract

**Background:**

Osteoarthritis (OA) contributes to heightened pain perception by disrupting the normal function of peripheral nerves and spinal nociceptive circuits. Although selective cyclooxygenase‐2 (COX‐2) inhibitors reduce OA‐associated pain, the distinct roles of spinal COX‐2 and glial cell activity in this context remain poorly defined.

**Methods:**

The effects of two COX‐2 inhibitors, etoricoxib and celecoxib, were examined using an anterior cruciate ligament transection (ACLT) rat model of OA. Mechanical allodynia was assessed behaviorally using the von Frey filament test. COX‐2 protein expression and glial cell (astrocyte and microglia) activation in the lumbar spinal cord were analysed via immunohistochemistry.

**Results:**

Spinal COX‐2 expression was significantly increased, mainly in neurons and astrocytes, at the 16th week after ACLT (*p* = 0.026). Microglia and astrocytes were activated from the 2nd to 16th week and from the 6th to 16th week after ACLT, respectively. The intrathecal median effective dose (ED_50_) of COX‐2 inhibitors, etoricoxib and celecoxib, required for reducing mechanical allodynia was lower at the 16th week than at the 2nd week after ACLT surgery (*p* = 0.0448 and 0.046, respectively). In contrast, the oral ED_50_ values of etoricoxib and celecoxib for relieving mechanical allodynia were slightly higher at the 16th week than at the 2nd week after ACLT surgery (*p* = 0.097 and 0.227, respectively).

**Conclusions:**

Our study shows that the efficacy of COX‐2 inhibitors in ALCT‐induced OA rats depends on the timing and route of administration. In the later phase, spinal glia cells exhibited increased activity and elevated COX‐2 expression.

**Significance:**

This study characterises the complex mechanisms underlying OA pain, involving both peripheral and central components, and highlights the stage‐specific involvement of COX‐2, particularly in the spinal cord. It provides experimental evidence linking central COX‐2 activity and glial cell responses to OA pain, offering insights into the temporal dynamics of pain processing and guiding the development of therapeutic strategies.

**Abbreviations**
ACLT: anterior cruciate ligament transection
aCSF: artificial cerebrospinal fluid
CCI: chronic constriction injury
COX: cyclooxygenase
CSF: cerebrospinal fluid
DMSO: dimethyl sulfoxide
GFAP: glial fibrillary acidic protein
i.t.: intrathecal
%MPE: percentage of the maximum possible effect
OA: osteoarthritis
OCT: optimal cutting temperature compound
PGE2: prostaglandin E2
PWT: hindpaw withdrawal threshold
SCDH: spinal cord dorsal horn

## 1 Introduction

Osteoarthritis (OA) is the most prevalent form of joint disease, with an estimated global incidence of 20% (Safiri et al. 2020). Pharmacological management of pain aims at reducing pain and functional impairment (Richard et al. 2023). The global OA pain therapeutics market reached US $7.93 billion in 2022, and is estimated to reach US $16.81 billion by 2030 (Growth Plus Reports 2022). Many patients with OA continue to experience pain despite receiving standard therapies targeting the joints, including joint‐replacement surgery (Cao et al. 2020; Maqbool et al. 2021). OA pain involves both nociceptive and neuropathic mechanisms, with central mechanisms driven by spinal neuroinflammation that contributes to pain amplification (Ji et al. 2018; Ohashi et al. 2023). This neuroinflammatory process is characterised by the activation of microglia and astrocytes, and increased production of proinflammatory mediators, including prostaglandins (Miller and Malfait 2017; Vergne‐Salle and Bertin 2021). Activated glial cells synthesise prostaglandins, the release of which enhances dorsal horn neuronal excitability, facilitating nociceptive signalling (Gebicke‐Haerter et al. 1988; Guastadisegni et al. 1997; Myers et al. 2006).

Two major isoforms of cyclooxygenase (COX) are responsible for prostaglandin synthesis: in most tissues, COX‐1 continually produces low levels of prostaglandins; however, in inflammatory tissues, inducible COX‐2 increases prostaglandin synthesis by approximately 100‐fold (Harris 2013). Therefore, COX‐2 plays a more important role in the inflammatory response than COX‐1. Spinal COX‐2 and its products (prostaglandins) contribute to nociceptive modulation during peripheral inflammation in OA‐induced nociception (Arendt‐Nielsen et al. 2016). COX‐2 is a recommended target for managing OA‐associated pain (Nakata et al. 2018). Sagar et al. (2011) demonstrated activation of glial cells in a rodent model of OA. However, reports on the correlation between spinal COX‐2 expression and glial cell activation in OA‐induced nociception, and on the efficacy and optimal time of administering COX‐2 inhibitors via different approaches remain scarce.

It is known that the spinal cord dorsal horn (SCDH) plays a vital role in transmitting nociceptive signals to the brain under peripheral acute or chronic pain circumstances (Milligan and Watkins 2009). The primary objective of this study was to explore the role of COX‐2 in the SCDH in the development and persistence of OA‐related pain in rats after anterior cruciate ligament transection (ACLT). Additionally, we examined SCDH glial cells, including microglia and astrocytes, and COX‐2 expression at various post‐ACLT time points. We also assessed the potential antinociceptive effects of intrathecal and systemic (oral) administration of two specific COX‐2‐selective inhibitors, etoricoxib and celecoxib, in rats with ACLT‐induced OA. The study provides valuable insights into the optimal timing and administration rules for using COX‐2 inhibitors to manage OA‐associated nociception and elucidates the relationship between COX‐2 and glial cells in the central nervous system (CNS).

## 2 Methods

### 2.1 Animals

Male Wistar rats (290–315 g; BioLASCO Taiwan Co. Ltd., Taipei, Taiwan), six rats per group, were used in this study. They were housed in a temperature‐controlled (22°C ± 1°C) room under light‐cycle‐controlled conditions (12‐h light/12‐h dark) and provided free access to food and water. The study was approved by the National Sun Yat‐sen University Animal Care and Use Committee (approval number 10520) and conformed to the Care and Use of Animals guidelines of the American Physiology Society. For all surgeries and intrathecal (i.t.) injections, the rats were anaesthetised with isoflurane (2%) inhalation. To prevent infection from surgery, the rats received an intramuscular postoperative administration of cephalosporin (cefazolin; 0.17 g/kg). Every effort in the experimental design and sample collection was made to minimise the suffering and the number of rats used.

### 2.2 Induction of OA by ACLT

As described in previous studies (Wen et al. 2013, 2010; Yang et al. 2014), we performed ACLT on the right knee of rats, and did not operate on the left knee. After exposing the ACL, it was directly visualised and transected via the midsubstance. For sham‐operated rats, we exposed the ACL but did not transect it. After surgery, the rats were not immobilised and were allowed unrestricted cage activity. The rats were closely monitored for infections and other complications following surgery.

### 2.3 Experimental Groups and Preparation of the Two COX‐2 Inhibitors

We examined the time course of protein expression of COX‐2, glial fibrillary acidic protein (GFAP), an astrocyte‐specific marker, and OX‐42, a microglia‐specific marker, in the lumbar SCDH after ACLT‐induced OA. The rats were randomly assigned to different groups: a sham group that received arthrotomy without ACLT; a 2w group, in which rats were sacrificed at 2 weeks after ACLT; a 6w group, in which the rats were sacrificed at 6 weeks after ACLT; a 16w group, in which the rats were sacrificed 16 weeks after ACLT. For evaluating the analgesic effects of COX‐2 inhibitors in the ACLT‐induced OA pain test, rats were i.t. administered the two inhibitors at doses of 0.01, 0.1, 0.5, 1, 5, 10 and 20 μg/rat or orally administered at doses of 1.5, 3, 6 mg/kg on the 2nd and 16th week after ACLT. The COX‐2 inhibitors, etoricoxib was purchased from Santa Cruz Biotechnology (Santa Cruz, CA, USA) and celecoxib was purchased from Pharmacia Corp. (St. Louis, MO, USA). Etoricoxib and celecoxib were dissolved in 20% dimethyl sulfoxide (DMSO) in saline for oral administration. For i.t. administration, etoricoxib and celecoxib were dissolved in 20% DMSO in artificial cerebrospinal fluid (aCSF; 122.7 mM Cl^−^, 21.0 mM HCO_3_^−^, 2.5 mM HPO_4_^2−^, 151.1 mM Na^+^, 2.6 mM K^+^, 0.9 mM Mg^2+^, 1.3 mM Ca^2+^ and 3.5 mM dextrose).

### 2.4 Implantation of i.t. Catheters

Through the atlanto‐occipital membrane at the base of the rat's skull, we implanted an i.t. catheter (PE5 tubes: 9‐cm long, 0.008‐in. inner diameter, 0.014‐in. outer diameter; Spectranetics, Colorado Springs, CO, USA) into the spinal lumbar enlargement using a method described previously (Jean et al. 2009). For spinal drug injection, we externalised and fixed one end of the i.t. catheter to the cranial aspect of the head of the rat. Because the dead capacity of the i.t. catheter was 3.5 μL, we administered an i.t. aCSF flush (10 μL) to rats following all i.t. injections to ensure complete drug delivery. Five days after the implantation surgery, rats with i.t. catheters that showed gross neurological injury or had fresh blood in the cerebrospinal fluid (CSF) were excluded from subsequent experiments.

### 2.5 Nociceptive Behavioural Testing

To assess mechanical allodynia, we measured the hind paw withdrawal threshold (PWT; g) using calibrated von Frey filaments (Stoelting, Wood Dale, IL, USA) after placing each rat into each compartment of a clear plastic cage, which was placed on an elevated metal mesh floor. According to Chaplan's ‘up–down’ method described previously (Chaplan et al. 1994; Huang et al. 2012), we used a series of von Frey filaments of logarithmically incremental stiffness onto the midplantar region of the hind paw for evaluating the closest filament to the threshold of pain symptoms (licking or withdrawal) of rats. PWL data from i.t. or oral administration were transformed to a percentage of the maximum possible effect (%MPE) using the following formula: %MPE = (post‐drug response—baseline)/(cut‐off‐baseline) × 100. The post administration latency is the PWT measured after administration of the compound or aCSF, the baseline is the PWT measured immediately prior to administration, and the cutoff stimulation is 10 g (Lin et al. 2011).

### 2.6 Spinal Immunohistofluorescence Analysis

To reduce variations in immunohistofluorescence procedures, the lumbar spinal tissues from rats in the different groups were mounted into the same optimal cutting temperature (OCT) compound block and sectioned together using a cryostat at −30°C (HM550; Microm, Waldorf, Germany) for subsequent analysis according to a previously described method with some modifications (Huang et al. 2012; Jean et al. 2009). The spinal sections (10 μm thick) were incubated with a primary antibody—anti‐COX‐2 (1:200 dilution; catalogue no. 160106; Cayman Chemical, Ann Arbor, MI, US), anti‐OX‐42 (CD11b, microglial marker; 1:200 dilution; catalogue no. CBL1512; EMD Millipore, Temecula, CA, USA), anti‐glial fibrillary acidic protein (GFAP) (astrocytic marker; 1:200 dilution; catalogue no. MAB3402; EMD Millipore) or anti‐neuronal nuclei (NeuN) (neuronal marker; 1:500, Alexa Fluor 488 conjugated antibody; catalogue no. MAB377X, EMD Millipore) overnight at 4°C. The sections were then incubated with DyLight 549‐conjugated donkey anti‐rabbit IgG antibody (1:400 dilution; catalogue no. 711‐506‐152; Jackson ImmunoResearch Laboratories Inc., West Grove, PA, USA; red fluorescence) or Alexa Fluor 488‐labelled chicken anti‐mouse IgG antibody (1:400 dilution; catalogue no. A‐21200; Molecular Probes, Eugene, OR, USA; green fluorescence) for 40 min at 25°C. The stained sections were visualised using a Leica DM‐6000 CS fluorescence microscope (Leica Instruments Inc., Wetzlar, Germany) and photographed using a SPOT Xplorer Digital camera (Diagnostic Instruments Inc., Sterling Heights, MI, USA). The ImageJ software (National Institutes of Health, Bethesda, MD, USA) was used to measure the pixel values of the immunoreactive areas (using three spinal sections per rat). Spinal neurons are distributed in the superficial laminae (laminae I–III), which respond to nociceptive stimuli and participate in the nociception transmission pathway to the brain (Raposo et al. 2015). Therefore, the superficial laminae play a more important role in chronic pain than the deep laminae. Hence, we quantified the immunoreactivity of the targeted protein in the spinal superficial laminae using the method employed for rodent models of pain (Chen et al. 2014; Kawasaki et al. 2008; Raposo et al. 2015; Stokes et al. 2013). Immunofluorescence data are shown as the fold change from the sham‐operated group. To identify resting and activated microglia, we used the criteria used in a previous study (Hains and Waxman 2006), and to distinguish resting and activated astrocytes, we followed another criterion from a previous study (Martin et al. 2003). For double staining of COX‐2 and neuronal marker, we incubated the spinal sections with a mixture of anti‐COX‐2 and anti‐NeuN antibodies overnight at 4°C. The sections were then incubated with DyLight 549‐conjugated anti‐rabbit IgG antibody (1:400 dilution) for 40 min at room temperature. For double staining of COX‐2 and OX‐42 or GFAP, we incubated the spinal sections with a mixture of anti‐COX‐2 and anti‐OX‐42 or anti‐GFAP antibodies overnight at 4°C. The spinal sections were then incubated with a mixture of DyLight 549‐conjugated anti‐rabbit IgG antibody (1:400 dilution) and Alexa Fluor 488‐conjugated anti‐mouse IgG antibody (1:400 dilution) for 40 min at room temperature. Double immunofluorescence images were further examined and acquired using a Leica DM‐6000 CS fluorescence microscope equipped with a SPOT Xplorer Digital camera.

### 2.7 Data and Statistical Analysis

The sample size was determined based on previous studies employing the same experimental design in ACLT‐induced OA pain in rats (Wen et al. 2024; Zhao et al. 2017) and was further calculated using GPower 3.1, with conventional parameters (effect size = 0.3, type I error [*α*] = 0.05 and power = 0.8) (Faul et al. 2007). For the behavioural experiments, a sample size of six animals per group was estimated using GPower. A sample size of six rats per group, with three randomly selected sections analysed per animal, was also used for the immunofluorescence analysis. All data are presented as the means ± standard error of the mean (SEM). For statistical analysis, a one‐way analysis of variance (ANOVA) and *t*‐test were used to calculate the difference between the groups, followed by the Tukey post hoc test. A value of *p* < 0.05 was considered to indicate a statistically significant difference.

## 3 Results

### 3.1 ACLT Affects the Nociception Behaviour and Spinal Glia Expression in the Rats

**FIGURE 1:**
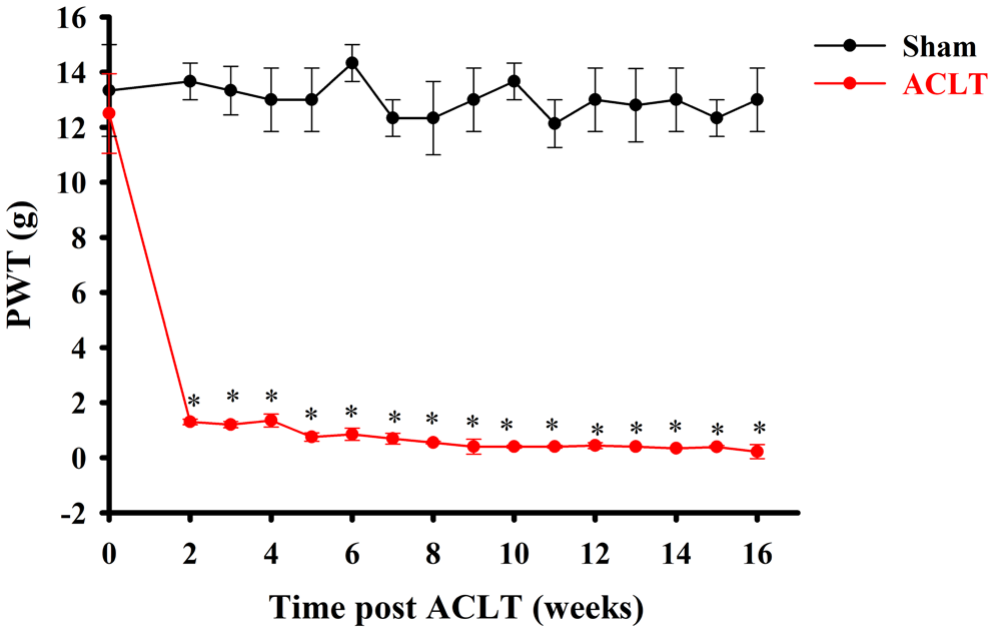
Time course of anterior cruciate ligament transection (ACLT)‐induced mechanical allodynia in rats. Mechanical allodynia was detected through the paw withdrawal threshold (PWT) every week after ACLT. *p < 0.05, compared with the sham group. Data are presented as mean ± SEM.

**FIGURE 2:**
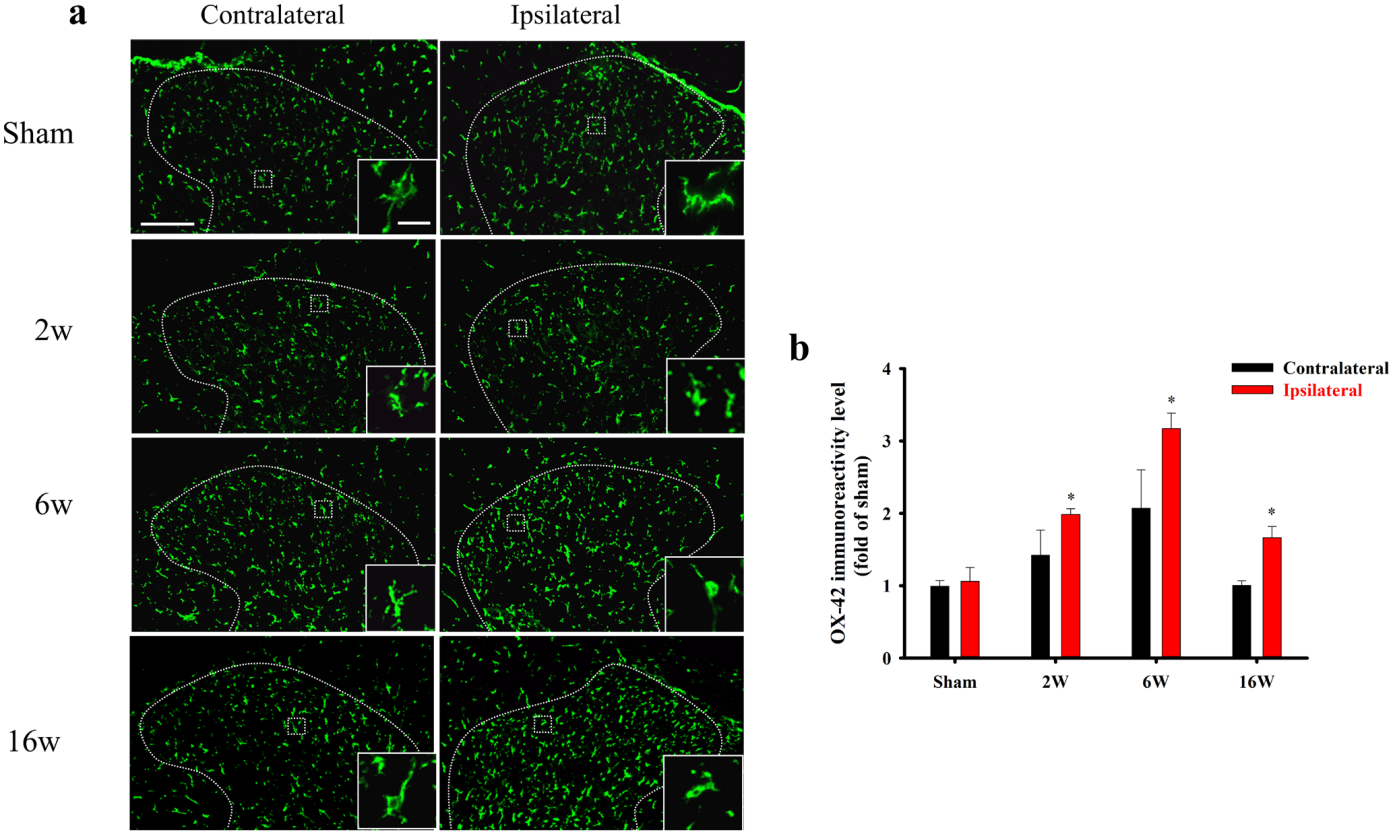
Anterior cruciate ligament transection (ACLT) activates the spinal microglia in rats. The lumbar spinal cord of rats in the sham and ACLT groups (2, 6, and 16 weeks of age) was harvested after ACLT. (a) A microglia‐specific marker (OX‐42, green) was used to visualise microglia in the spinal cord. High‐magnification images of OX‐42 expression in the ipsilateral and contralateral spinal dorsal horns are presented in the inset. (b) Quantification of OX‐42 in the ipsilateral and contralateral spinal dorsal horns, shown as means ± SEM. Spinal OX‐42 was upregulated by ACLT and expressed primarily in the ipsilateral spinal dorsal horn, especially at Week 6. Scale bars: 100 μm (a); 10 μm in the inset. *p < 0.05, compared with the sham group.

As shown in Figure 1, the sham group had a stable PWT for over 16 weeks. The mechanical allodynia induced by ACLT peaked at the 2nd week after surgery and persisted until at least the 16th week after surgery in the ACLT group. To explore the potential changes in the spinal microglia and astrocytes in ACLT rats, the lumbar spinal tissues were prepared from rats of all the groups. As evident from the immunohistochemistry images using the OX‐42 antibody, which labels microglial cells, the resident microglia from the sham‐operated rats had a resting‐type shape and small compact cell soma diameters with long and finely branched processes on both the contralateral and ipsilateral sides (Figure 2a, inset). On the ipsilateral side of the ACLT rats from 2nd to the 16th week after ACLT surgery, the microglia showed increased OX‐42 immunoreactivity and exhibited an activated state and enlarged hypertrophic soma with retraction of cytoplasmic processes (Figure 2a, inset). Compared with the sham‐operated group, ACLT‐induced microglial activation was densest in laminae I–III within the dorsal horn on the ipsilateral side from the 2nd week to the 16th week. Quantification of OX‐42 immunoreactivity indicated that ACLT‐induced OA significantly upregulated OX‐42 immunoreactivity on the ipsilateral side at the 2nd, 6th and 16th weeks after surgery (*p* = 0.018, < 0.001 and 0.01, respectively, Figure 2b).

**FIGURE 3:**
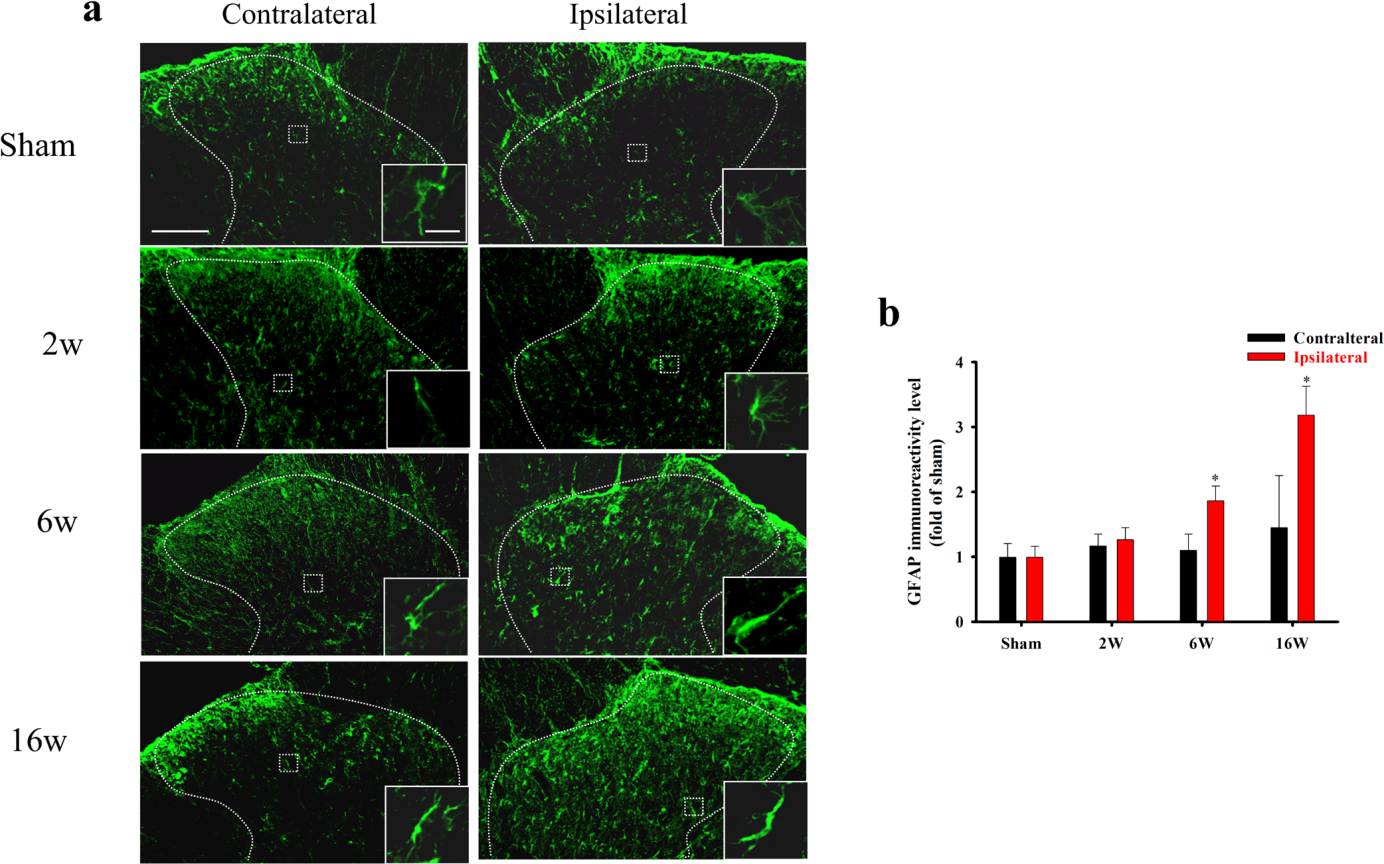
Anterior cruciate ligament transection (ACLT) activates the spinal astrocytes in rats. The lumbar spinal cord of rats in the sham and ACLT groups (2, 6, and 16 weeks of age) was harvested after ACLT. (a) An astrocyte‐specific marker (GFAP, green) was used to visualise the astrocytes in the spinal cord. High‐magnification images of GFAP expression in the ipsilateral and contralateral spinal dorsal horns are presented in the inset. (b) Quantification of GFAP in the ipsilateral and contralateral spinal dorsal horns is shown as means ± SEM. Spinal GFAP was upregulated by ACLT and expressed primarily in the ipsilateral spinal dorsal horn, especially at Weeks 6 and 16. Scale bars: 100 μm (a); 10 μm in the inset. *p < 0.05, compared with the sham group.

Immunohistochemical labeling of astrocytes using the anti‐GFAP antibody revealed that the resident astrocytes from sham‐operated rats had a resting‐type shape and thin soma, with long and slender processes on the contralateral and ipsilateral sides (Figure 3a, inset). On the ipsilateral side of the ACLT rats from the 2nd to the 16th week after surgery, astrocytes exhibited increased GFAP immunoreactivity and appeared to be in an activated state, with hypertrophied soma and numerous thick processes. A significant increase in spinal GFAP immunoreactivity in the ipsilateral dorsal grey matter was evident from the 6th to the 16th week after surgery. Quantification of GFAP immunoreactivity also showed that OA induced by ACLT significantly upregulated GFAP immunoreactivity on the ipsilateral side from the 6th to the 16th weeks after surgery (*p* = 0.0327 and < 0.001, respectively, Figure 3b). Collectively, ACLT would induce microglia and astrocytes activation after ACLT surgery, and the sham surgery would not affect the expression of glial cells in the SCDH.

### 3.2 Effect of ACLT‐Induced OA on Spinal COX‐2 Expression

**FIGURE 4:**
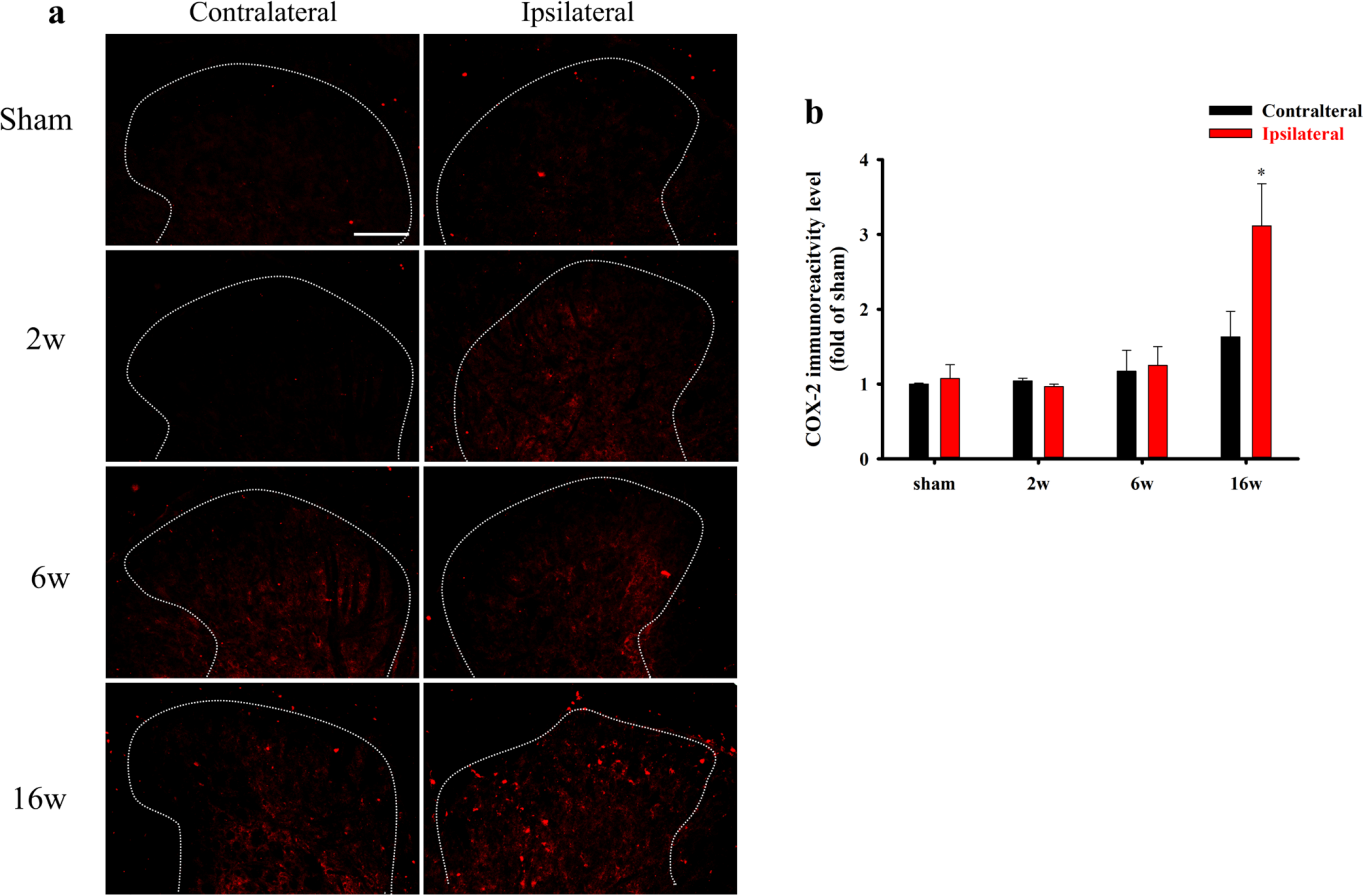
Spinal COX‐2 expression in the rats subjected to anterior cruciate ligament transection (ACLT). The lumbar spinal cord of rats in the sham and ACLT groups (2, 6, and 16 weeks of age) was harvested after ACLT. (a) COX‐2 expression is presented in the ipsilateral and contralateral spinal cord dorsal horns in the sham, 2w, 6w and 16w groups. (b) Quantification of COX‐2 expression in the ipsilateral and contralateral spinal dorsal horns is shown as means ± SEM. ACLT significantly upregulated COX‐2 immunoreactivity on the ipsilateral side at 16 weeks after surgery. Scale bar: 100 μm. *p < 0.05, compared with the sham group.

Basal levels of spinal COX‐2 were detectable in both the contralateral and ipsilateral dorsal horns of rats in the sham‐operated group (Figure 4a). Compared with the sham‐operated group, COX‐2 immunoreactivity was clearly increased on the ipsilateral side at the 16 week after surgery, which was confirmed by quantification of COX‐2 immunoreactivity (*p* = 0.003, Figure 4b).

**FIGURE 5:**
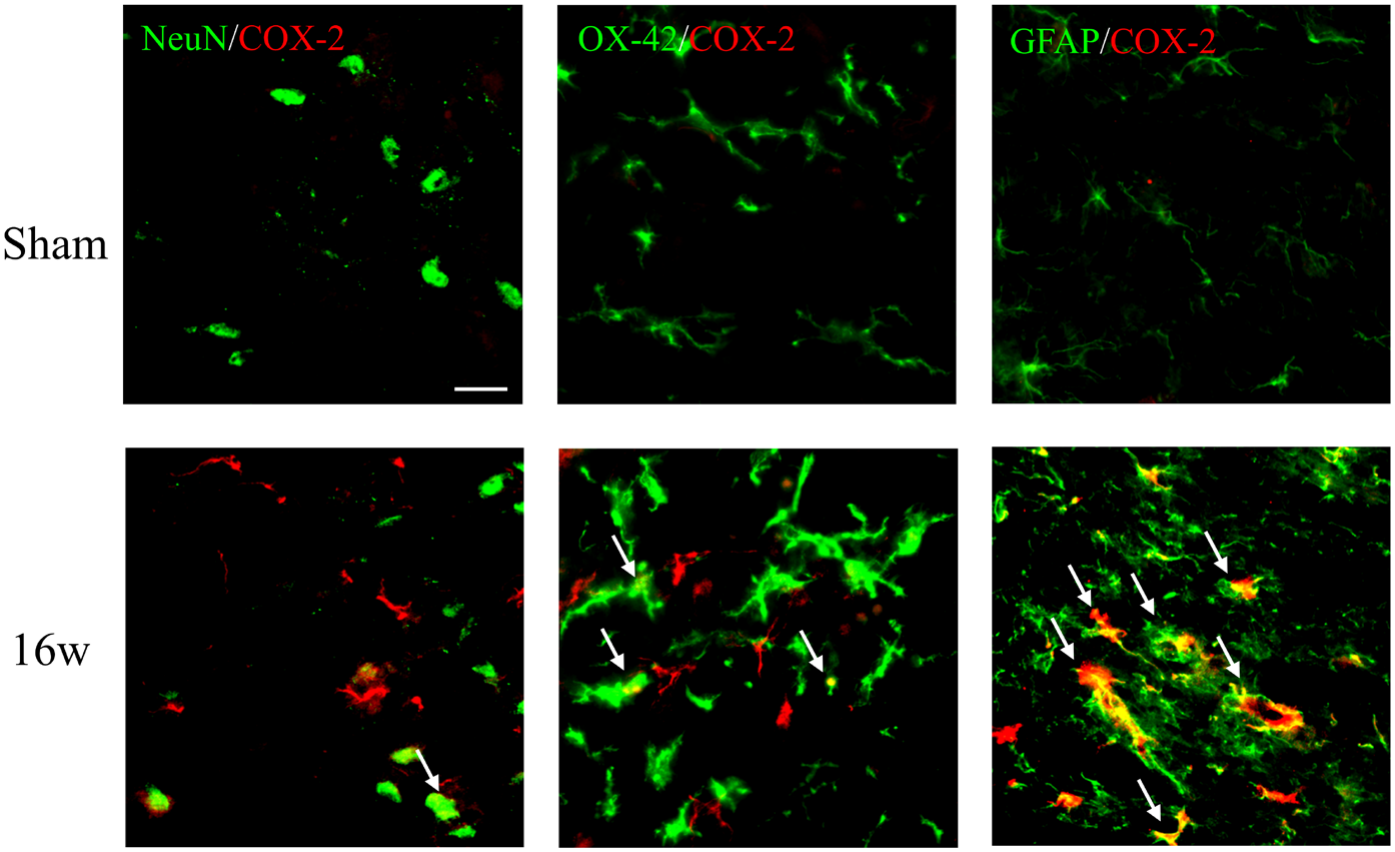
Spinal COX‐2 is expressed in specific cells in the late phase after anterior cruciate ligament transection (ACLT). The cellular specificity of COX‐2 expression in the dorsal region of the lumbar spinal cord dorsal horn was ipsilateral to the injury site after ACLT surgery in the 16th week. Double‐immunofluorescence staining of COX‐2 (red) with NeuN (a neuron‐specific marker, green), OX‐42 (a microglia‐specific marker, green), or GFAP (an astrocyte‐specific marker, green) in the spinal cord from rats in the sham and 16w groups. The merged images show that COX‐2 expression was lower in the sham‐operated group, but its was highly expressed in the 16w group and colocalized with the three cell‐specific markers. Spinal COX‐2 was primarily colocalized with astrocytes rather than with neurons and microglia in ACLT rats in the 16th week. Scale bar: 50 μm.

Additionally, no increasing trend in COX‐2 immunoreactivity was noted on either the contralateral or ipsilateral sides from the 2nd to the 6th week after surgery. To identify which cell types colocalized with the upregulation of COX‐2 expression after ACLT, we performed double immunofluorescence staining of COX‐2 (red) with neuron‐specific marker (NeuN, green), microglia‐specific marker (OX‐42, green) and astrocyte‐specific marker (GFAP, green) (Figure 5). In the sham group, COX‐2 was less colocalized with neurons, microglia and astrocytes. However, all three cell‐specific markers (NeuN, OX‐42 and GFAP) colocalized with COX‐2 in the 16w group. Furthermore, the upregulation of COX‐2 was mainly evident in astrocytes. These observations indicated that OA induced by ACLT caused the upregulation of COX‐2, which mainly colocalized with astrocytes rather than neuronal or microglial cells at the 16th week after surgery.

### 3.3 Effect of i.t. Administration of COX‐2‐Selective Inhibitors on Nociception During the Early and Late Phases of OA

**FIGURE 6:**
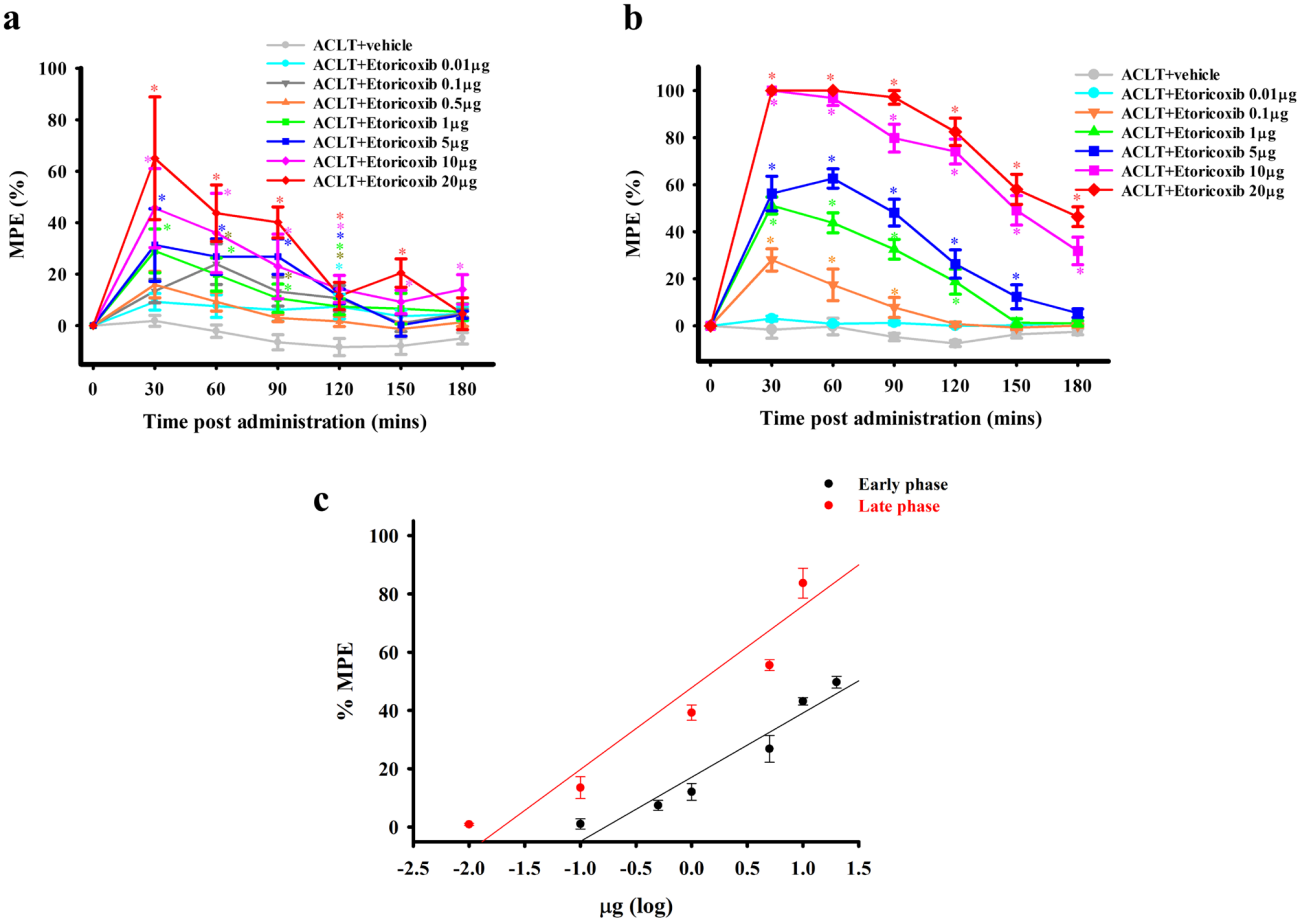
Effect of intrathecal administration of etoricoxib on antinociception during the early and late phases in rats subjected to anterior cruciate ligament transection (ACLT). Time course effects of the % maximum possible effect (MPE) of anti‐mechanical allodynia for intrathecal injection of etoricoxib in ACLT‐rats at 2 (a) and 16 (b) weeks after ACLT. In (a, b), the horizontal axis represents the time in minutes after intrathecal injection, and the vertical axis represents the %MPE, calculated as the mean for six animals per dose. Data in (a, b) were transformed to a dose–response curve of etoricoxib for anti‐mechanical allodynia (c). The ED_50_ values of etoricoxib at 2 and 16 weeks after surgery were 4.36 ± 1.16 and 1.49 ± 0.47 μg, respectively. The anti‐mechanical allodynia effect of intrathecal etoricoxib administration was higher at 16 weeks after surgery than at 2 weeks after surgery. *p < 0.05, compared with the ACLT + vehicle group.

We examined the antinociceptive effects of i.t. administration of clinical COX‐2‐selective inhibitors (etoricoxib and celecoxib) on ACLT‐induced mechanical allodynia during the early (the 2nd week) and late (the 16th week) phases after surgery. Intrathecal (i.t.) administration of etoricoxib produced a significant, dose‐dependent antinociceptive effect, as reflected by an increased percentage of maximal possible effect (%MPE) in the treatment groups compared with that in the ACLT + vehicle group during both the early and late phases (Figure 6a,b). For ease of analysis, the data were transformed to a dose–response curve of the average %MPE from 30 to 180 min after i.t. etoricoxib administration (Figure 6c). The median effective doses (ED_50_) for antinociceptive effect of i.t. etoricoxib administration in the early and late phase after surgery were 4.36 ± 1.16 and 1.49 ± 0.47 μg, respectively. The anti‐mechanical allodynia effect of i.t. administration of etoricoxib was significantly better in the late phase than that for the early phase of ACLT‐induced OA pain (*p* = 0.0448).

**FIGURE 7:**
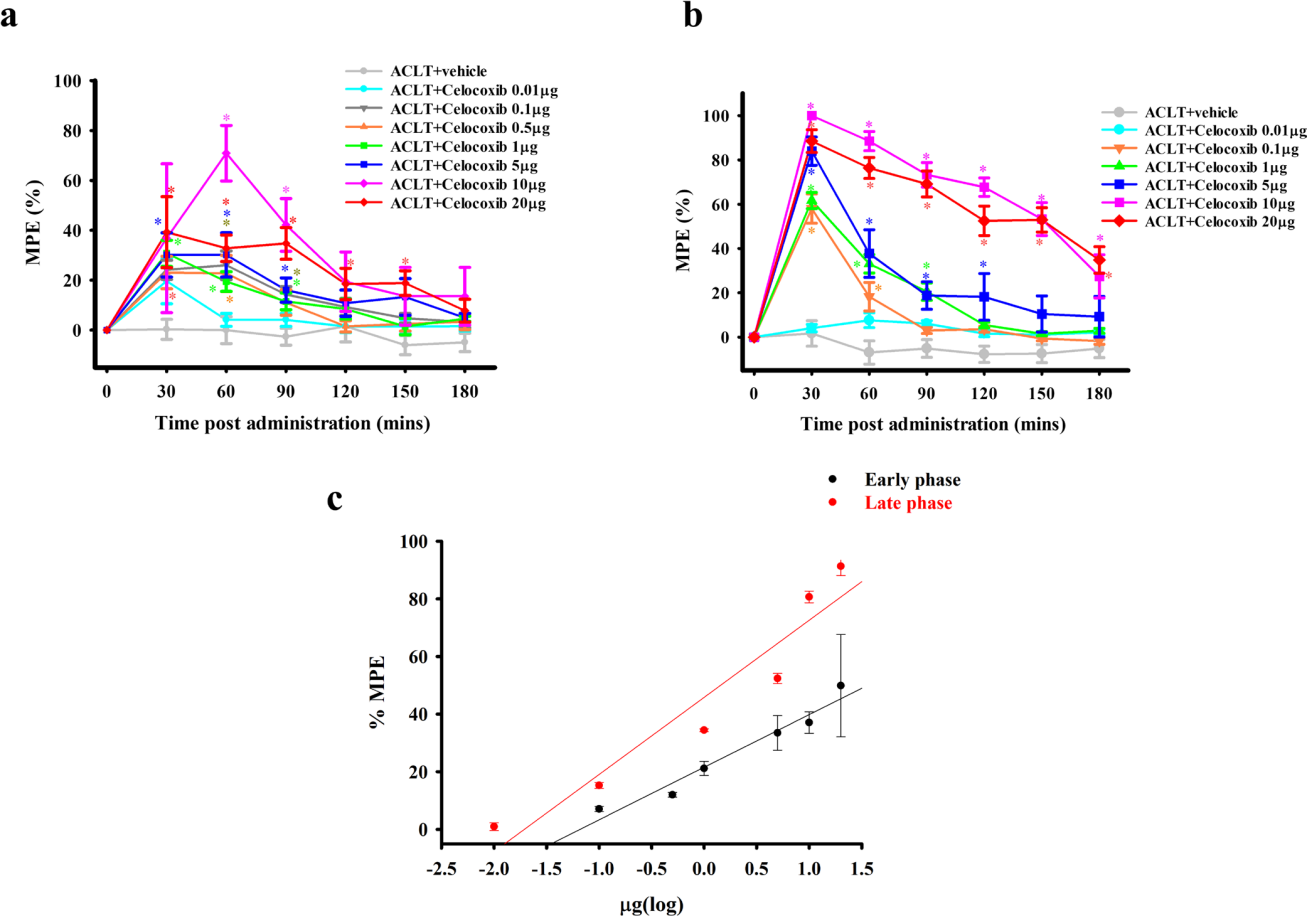
Effect of intrathecal administration of celecoxib on antinociception during the early phase and late phases in rats subjected to anterior cruciate ligament transection (ACLT). Time course effects of the % maximum possible effect (MPE) of anti‐mechanical allodynia for intrathecal injection of celecoxib in ACLT‐rats 2 (a) and 16 (b) weeks after ACLT. In (a, b), the horizontal axis represents the time in minutes after intrathecal injection, and the vertical axis represents the %MPE, calculated as the mean for six animals per dose. Data in (a, b) were transformed to a dose–response curve of etoricoxib for anti‐mechanical allodynia (c). The ED_50_ values of etoricoxib at 2 and 16 weeks after surgery were 9.60 ± 5.04 and 2.55 ± 0.54 μg, respectively. The anti‐mechanical allodynia effect of intrathecal celecoxib administration was higher at 16 weeks after surgery than at 2 weeks after surgery. *p < 0.05, compared with the ACLT + vehicle group.

I.t. administration of another clinical COX‐2‐selective inhibitor, celecoxib, also produced an apparent dose‐dependent antinociceptive effect (%MPE) in the treatment groups in the early and late phases compared with that in the ACLT + vehicle group (Figure 7a,b). These data were transformed to a dose–response curve of etoricoxib for antinociception (Figure 7c). The ED_50_ values for anti‐mechanical allodynia effects of i.t. celecoxib administration in the early and late phases were 9.60 ± 3.05 and 2.55 ± 0.54 μg, respectively. Similar to etoricoxib, the anti‐mechanical allodynia effect of i.t. celecoxib administration was also significantly better in the late phase than in the early phase (*p* = 0.046).

### 3.4 Effect of Oral Administration of COX‐2‐Selective Inhibitors on Nociception During the Early and Late Phases of OA

**FIGURE 8:**
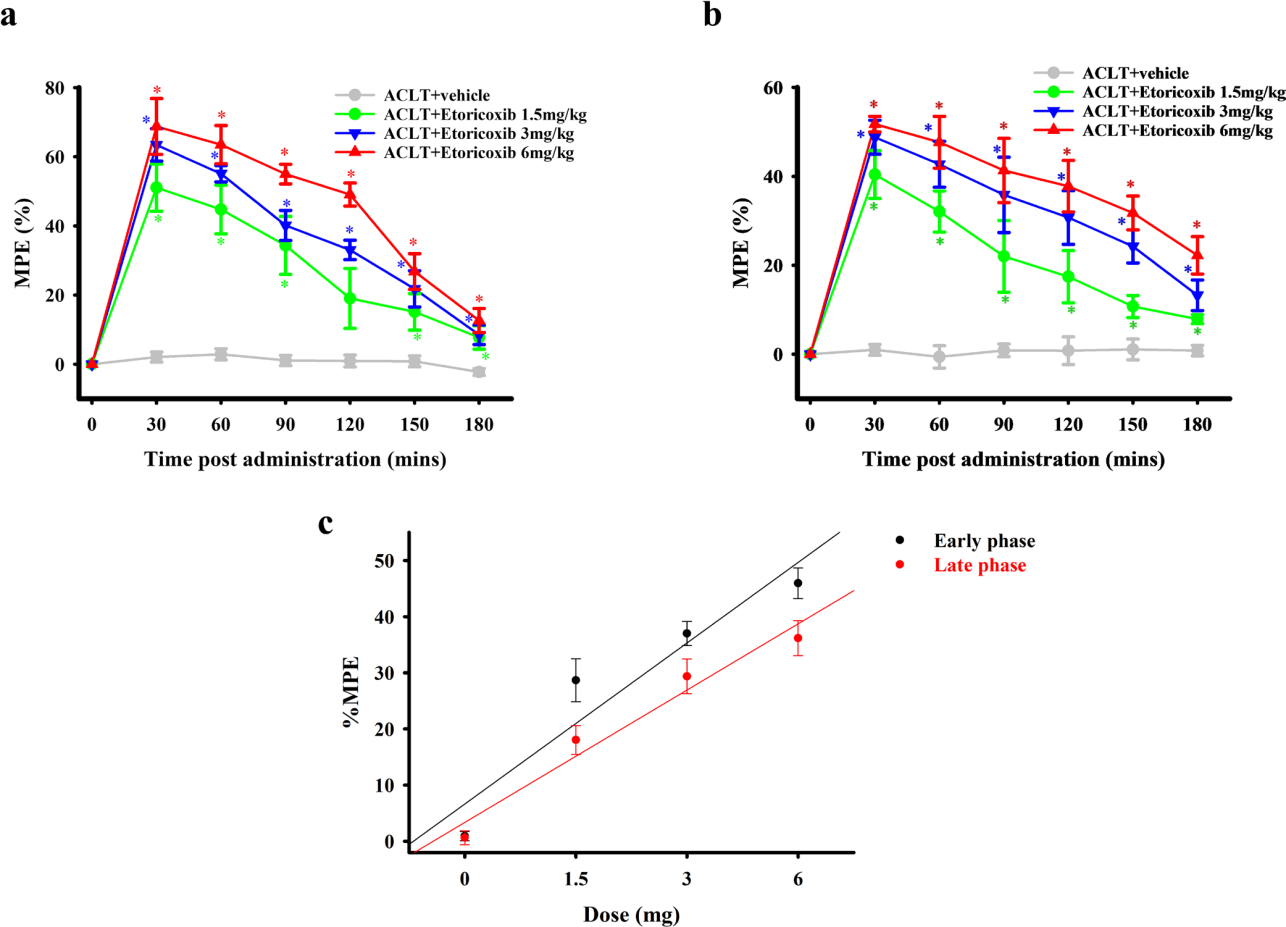
Effect of oral administration of etoricoxib on nociception during the early and late phases in rats subjected to anterior cruciate ligament transection (ACLT). Time course effects of the % maximum possible effect (MPE) of anti‐mechanical allodynia for oral administration of etoricoxib in ACLT‐rats at 2 (a) and 16 (b) weeks after ACLT. In (a, b), the horizontal axis represents the time in minutes after oral administration, and the vertical axis represents the %MPE, calculated as the mean for six animals per dose. Data in (a, b) were transformed to a dose–response curve of etoricoxib for anti‐mechanical allodynia (c). The ED_50_ values of etoricoxib at 2 and 16 weeks after surgery were 1.55 ± 0.2 and 2.69 ± 0.59 mg, respectively. The anti‐mechanical allodynia effect of oral etoricoxib administration was higher at 16 weeks after surgery than at 2 weeks after surgery. *p < 0.05, compared with the ACLT + vehicle group.

We further examined the antinociceptive effects of the systemic oral administration of etoricoxib and celecoxib on ACLT‐induced mechanical allodynia during the early (the 2nd week) and late (the 16th week) phases after surgery. Based on the well‐known hypothesis that the systemic dosage is approximately 100‐times that of the central dosage (Sindt et al. 2021), and considering our results for the i.t. administration of etoricoxib and celecoxib on nociception in OA rats (Figures 6 and 7), we selected three oral doses of etoricoxib and celecoxib (1.5, 3 and 6 mg/kg body weight). Oral administration of etoricoxib produced a significant, dose‐dependent antinociceptive effect, as reflected by an increased %MPE in the treatment groups compared with that in the ACLT + vehicle group during both the early and late phases (Figure 8a,b). For ease of analysis, the data were transformed to a dose–response curve of the average %MPE from 30 to 180 min after oral etoricoxib administration (Figure 8c). The ED_50_ for the antinociceptive effect of oral etoricoxib in the early and late phases were 1.55 ± 0.2 and 2.69 ± 0.59 mg, respectively (*p* = 0.097).

**FIGURE 9:**
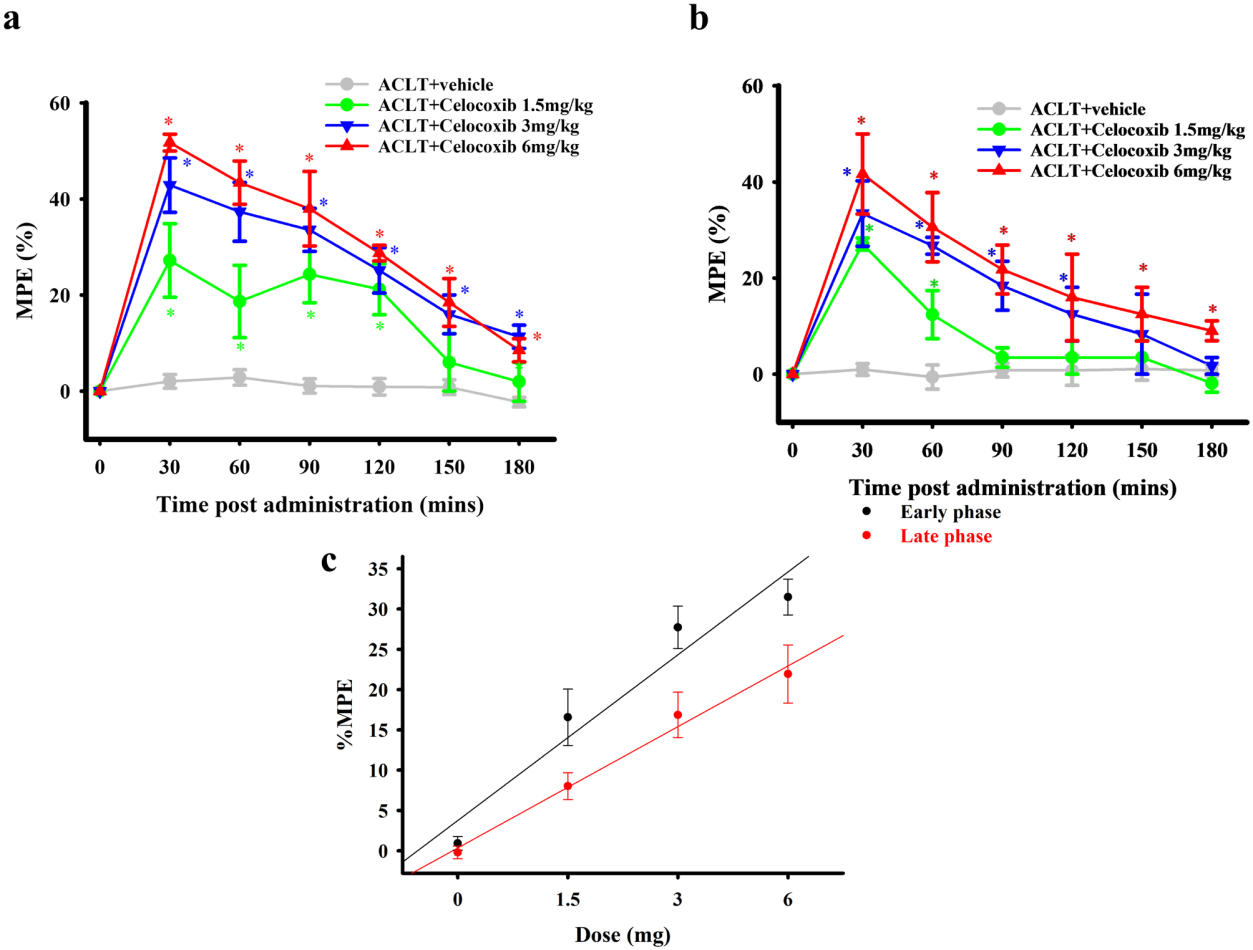
Effect of oral administration of celecoxib on nociception during the early phase and the late phase in rats subjected to anterior cruciate ligament transection (ACLT). Time course of the % maximum possible effect (MPE) of anti‐mechanical allodynia for oral administration of celecoxib in ACLT‐rats at 2 (a) and 16 (b) weeks after ACLT. In (a, b), the horizontal axis represents the time in minutes after oral administration, and the vertical axis represents the %MPE, calculated as the mean for six animals per dose. Data in (a, b) were transformed to a dose–response curve of celecoxib for anti‐mechanical allodynia (c). The ED_50_ values of celecoxib at 2 and 16 weeks after surgery were 2.17 ± 0.82 and 5.17 ± 2.48 mg, respectively. The anti‐mechanical allodynia effect of oral celecoxib administration was higher at 16 weeks after surgery than at 2 weeks after surgery. *p < 0.05, compared with the ACLT + vehicle group.

Oral administration of celecoxib produced an apparent antinociceptive effect in the treatment groups in the early and late phases compared with that in the ACLT + vehicle group (Figure 9a,b). To simplify data analysis, the data were transformed to a dose–response curve of the average %MPE from 30 to 180 min after oral celecoxib administration (Figure 9c). The ED_50_ for the antinociceptive effect of oral celecoxib in the early and late phases were 2.17 ± 0.82 and 5.17 ± 2.48 mg, respectively (*p* = 0.227). Thus, the anti‐mechanical allodynia effects of the oral administration of etoricoxib and celecoxib were slightly better in the early phase than in the late phase.

## 4 Discussion

In this study, we explored the involvement of spinal COX‐2 in the development and maintenance of OA pain in rats that underwent ACLT. We found that ACLT‐induced OA elicited mechanical allodynia in rats, starting from the 2nd week and continuing until the 16th week. Furthermore, we noted sustained activation of microglia and astrocytes in the ipsilateral lumbar SCDH, spanning week 2–16 and week 6–16 post‐ACLT surgery, respectively. Notably, upregulation of COX‐2 in the spinal cord was observed during the late phase (week 16) but not in the early phase of ACLT‐induced OA. Double‐immunostaining analysis confirmed that spinal astrocytes predominantly contributed to COX‐2 expression compared with neurons and microglia on the 16th week after ACLT. Additionally, the anti‐mechanical allodynia effects of orally administered etoricoxib and celecoxib were superior in the early phase compared to those in the late phase of OA. Importantly, neither i.t. nor oral administration of etoricoxib or celecoxib resulted in any discernible abnormal external behaviour or neurological dysfunction in the ACLT rats. Our findings suggest that spinal COX‐2 and neuroinflammation play a pivotal role in the perpetuation of long‐term chronic pain associated with OA.

Clinical nociceptive syndrome is characterised by evoked pain such as allodynia (Baron 2009). We found that ACLT caused mechanical allodynia that was well‐developed by 2 weeks and persisted for at least 16 weeks in rats after the surgery (Figure 1), which is also consistent with the results of previous studies (Wen et al. 2020, 2013, 2010). Central sensitization, a persistent pathological process in the CNS (Woller et al. 2017), is implicated in chronic pain conditions such as OA (Adães et al. 2014; Im et al. 2010). It involves heightened spontaneous neuronal activity, reduced activation thresholds, and expanded receptive fields, and leads to symptoms such as allodynia and hyperalgesia (Ji et al. 2018). The key mechanisms underlying central sensitization include neuroinflammation mediated by cytokines and neurotransmitters, as well as activation of nociceptive neurons in spinal cord lamina I–III (Benarroch 2016; Gu et al. 2016). Peripheral inflammation contributes to this central sensitization, as evident in OA models, wherein increased expression of TRPV1 and CGRP, elevated proinflammatory cytokines, and glial activation (microglia and astrocytes) are observed (Adães et al. 2015; Im et al. 2010; Ji et al. 2018). This glial activation is closely associated with chronic pain development and maintenance, and its inhibition has potential analgesic effects (Cao and Zhang 2008). Additionally, COX‐2 upregulation enhances neuronal excitability in the spinal cord, promoting hyperalgesia (Seybold et al. 2003), and COX‐2 inhibitors may be effective in mitigating central sensitization linked to peripheral inflammation (Woolf 2011).

In a previous study, intra‐articular injection of monoiodoacetate (MIA) induced mechanical allodynia, thermal hyperalgesia, and upregulation of COX‐2 protein expression in the ipsilateral spinal cord of rats (Prochazkova et al. 2009). Bove et al. reported that COX‐2 inhibition alleviated OA pain in rats following medial meniscal tear surgery (Bove et al. 2006). Tran and colleagues demonstrated that destabilisation of the medial meniscus‐induced OA in mice enhanced microglial activation in the SCDH (Tran et al. 2017). Intra‐articular injection of collagenase or MIA has been shown to elicit nociceptive responses and to upregulate astrocyte activation and microglial marker expression in the ipsilateral SCDH (Adães et al. 2017; Sagar et al. 2011). Despite these findings, the temporal dynamics of COX‐2 expression and its interaction with glial cells in the spinal cord under experimental OA models conditions remain incompletely characterised. In the present study, we found a significant upregulation of COX‐2 expression at the 16th week post‐ACLT surgery, which is similar to previous findings (Prochazkova et al. 2009), with the protein localising to both neurons and astrocytes. Notably, microglial and astrocytic activation in the ipsilateral SCDH was markedly increased from 2 to 16 weeks and from 6 to 16 weeks, respectively, following ACLT surgery. These findings indicate a time‐dependent glial response in OA‐induced nociception. The observed pattern of microglial activation aligned with previous reports from MIA‐induced OA models (Miller et al. 2013; Ogbonna et al. 2013), and the time course of astrocytic activation was consistent with that described by Sagar et al. (2011) but differed from that described by Ogbonna et al. (2013), suggesting potential model‐specific or experimental variability. Our previous studies (Huang et al. 2015; Lin et al. 2011), along with the present findings, support the hypothesis that activation of spinal glia contributes to the persistence of OA‐associated nociception. Notably, microglial activation peaked at the 6th week post‐ACLT, whereas astrocytic activation reached its maximum at the 16th week. These temporal differences support the notion that microglia primarily contribute to the initiation or development of chronic pain, whereas astrocytes may be more involved in its maintenance (Padi and Kulkarni 2008; Zhuang et al. 2006). Collectively, these findings provide novel insights into the temporal regulation of COX‐2 expression and glial cell activation in the ipsilateral SCDH, advancing our understanding of central mechanisms underlying OA‐induced nociceptive processing.

Previous studies have shown that COX‐2 expression regulates prostaglandin E2 (PGE2) levels in the chondrocytes of human OA cartilage (Amin et al. 1997; Fei et al. 2019). Peripheral administration of selective COX‐2 inhibitors can attenuate OA progression in animal models (Wen et al. 2020, 2013). Moreover, the systemic administration of selective COX‐2 inhibitors inhibited OA‐induced chronic nociceptive sensitisation in the previous studies (Arendt‐Nielsen et al. 2016). Systemic administration of COX‐2 selective inhibitors is one of the most commonly used clinical treatments for chronic pain in OA. However, central COX‐2 plays a critical role in nociception in chronic peripheral pain. To the best of our knowledge, only a few studies have suggested that spinal COX‐2 affects OA pain.

COX‐2 is a well‐established proinflammatory mediator implicated in both neuropathic and inflammatory pain. It is associated with spinal microglia during neuropathic pain (Yin et al. 2019) and is highly expressed in LPS‐stimulated astrocytes (Font‐Nieves et al. 2012). In peripheral inflammation, COX‐2 drives the synthesis of prostaglandin E2 (PGE2), a key contributor to acute and chronic pain (Kim 2014; Mollace et al. 2005). PGE2 also maintains neuropathic pain (Kawabata 2011), suggesting a role for spinal COX‐2‐mediated PGE2 in chronic OA pain. Although some studies suggest spinal COX‐2 primarily contributes to pain development (Takeda et al. 2005; Zhao et al. 2000), our findings indicate it may also be involved in pain maintenance during late‐stage OA. However, the identity of spinal cell types that express COX‐2 and mediate nociceptive transmission after peripheral injury‐induced chronic pain remains controversial. Our study is the first to demonstrate COX‐2 expression in astrocytes and neurons within the ipsilateral lumbar SCDH under ACLT‐induced OA, particularly during the late phase. We further examined whether central and peripheral treatment with the clinical COX‐2‐selective inhibitors (etoricoxib and celecoxib) affected nociception in the early and late phases of OA in rats, respectively. The antinociceptive ED_50_ of the i.t. administration of etoricoxib and celecoxib in the late phase of ACLT‐induced pain was lower than that in the early phase. Furthermore, the antinociceptive ED_50_ of the orally administered etoricoxib and celecoxib in the early phase of ACLT‐induced pain was lower than that in the late phase. Our findings further indicate that spinal COX‐2 contributes to the maintenance of OA pain rather than its development.

Although COX‐2 expression in OA cartilage and the analgesic effects of peripheral COX‐2 inhibitors are well documented (Arendt‐Nielsen et al. 2016; Fei et al. 2019; Wen et al. 2013), the central role of COX‐2 in OA pain has received less attention. Spinal COX‐2‐derived PGE2 is known to induce nociception (Vanegas and Schaible 2001), and intrathecal PGE1/PGE2 produces long‐lasting allodynia (Saito et al. 1995; Vanegas and Schaible 2001). These findings support our hypothesis that spinal COX‐2 contributes to the persistence of OA pain, potentially explaining why patients often experience pain despite effective joint‐targeted therapies (Hassan and Walsh 2014).

## 5 Conclusions

In this study, we discovered that COX‐2 expression in the ipsilateral SCDH was not elevated during the early phase after ACLT surgery but was significantly upregulated in the late phase. Notably, the route of COX‐2 inhibitor administration—intrathecal versus oral—was associated with distinct analgesic outcomes depending on the disease stage. In particular, intrathecal administration produced superior analgesic effects in the late phase, whereas oral administration was more effective during the early phase. These findings provide preclinical evidence supporting the potential of combined peripheral inhibition and central modulation strategies for improving the management of chronic OA pain.

## Author Contributions

Chun‐Sung Sung and Zhi‐Hong Wen participated in the conception and design of the study, data collection, interpretation of results and manuscript writing and critical revision. Shi‐Ying Huang participated in data collection, interpretation of results, manuscript writing and critical revision. Hao‐Jung Cheng and Sung‐Chun Lin participated in the conception and design of the study, data collection and interpretation of the results. Zong‐Sheng Wu contributed to the diagram. Zhi‐Kang Yao and Nan‐Fu Chen participated in manuscript review. Yen‐Hsuan Jean participated in the conception and design of the study and in research funding. All authors discussed the results and commented on the manuscript.

## Ethics Statement

The study was approved by the National Sun Yat‐sen University Animal Care and Use Committee (approval number 10520) and conformed to the Care and Use of Animals guidelines of the American Physiological Society and ARRIVE.

## Conflicts of Interest

The authors declare no conflicts of interest.
